# Dysregulation of lysine acetylation in the pathogenesis of digestive tract cancers and its clinical applications

**DOI:** 10.3389/fcell.2024.1447939

**Published:** 2024-09-26

**Authors:** Penghui Li, Yuan Xue

**Affiliations:** ^1^ Department of Gastrointestinal surgery, The First Affiliated Hospital, College of Clinical Medicine, Henan University of Science and Technology, Luoyang, Henan, China; ^2^ Department of thyroid surgery, The First Affiliated Hospital, College of Clinical Medicine, Henan University of Science and Technology, Luoyang, Henan, China

**Keywords:** lysine acetylation, digestive tract cancers, expression, pathogenic mechanisms, clinical application

## Abstract

Recent advances in high-resolution mass spectrometry-based proteomics have improved our understanding of lysine acetylation in proteins, including histones and non-histone proteins. Lysine acetylation, a reversible post-translational modification, is catalyzed by lysine acetyltransferases (KATs) and lysine deacetylases (KDACs). Proteins comprising evolutionarily conserved bromodomains (BRDs) recognize these acetylated lysine residues and consequently activate transcription. Lysine acetylation regulates almost all cellular processes, including transcription, cell cycle progression, and metabolic functions. Studies have reported the aberrant expression, translocation, and mutation of genes encoding lysine acetylation regulators in various cancers, including digestive tract cancers. These dysregulated lysine acetylation regulators contribute to the pathogenesis of digestive system cancers by modulating the expression and activity of cancer-related genes or pathways. Several inhibitors targeting KATs, KDACs, and BRDs are currently in preclinical trials and have demonstrated anti-cancer effects. Digestive tract cancers, including encompass esophageal, gastric, colorectal, liver, and pancreatic cancers, represent a group of heterogeneous malignancies. However, these cancers are typically diagnosed at an advanced stage owing to the lack of early symptoms and are consequently associated with poor 5-year survival rates. Thus, there is an urgent need to identify novel biomarkers for early detection, as well as to accurately predict the clinical outcomes and identify effective therapeutic targets for these malignancies. Although the role of lysine acetylation in digestive tract cancers remains unclear, further analysis could improve our understanding of its role in the pathogenesis of digestive tract cancers. This review aims to summarize the implications and pathogenic mechanisms of lysine acetylation dysregulation in digestive tract cancers, as well as its potential clinical applications.

## Background

**FIGURE 1:**
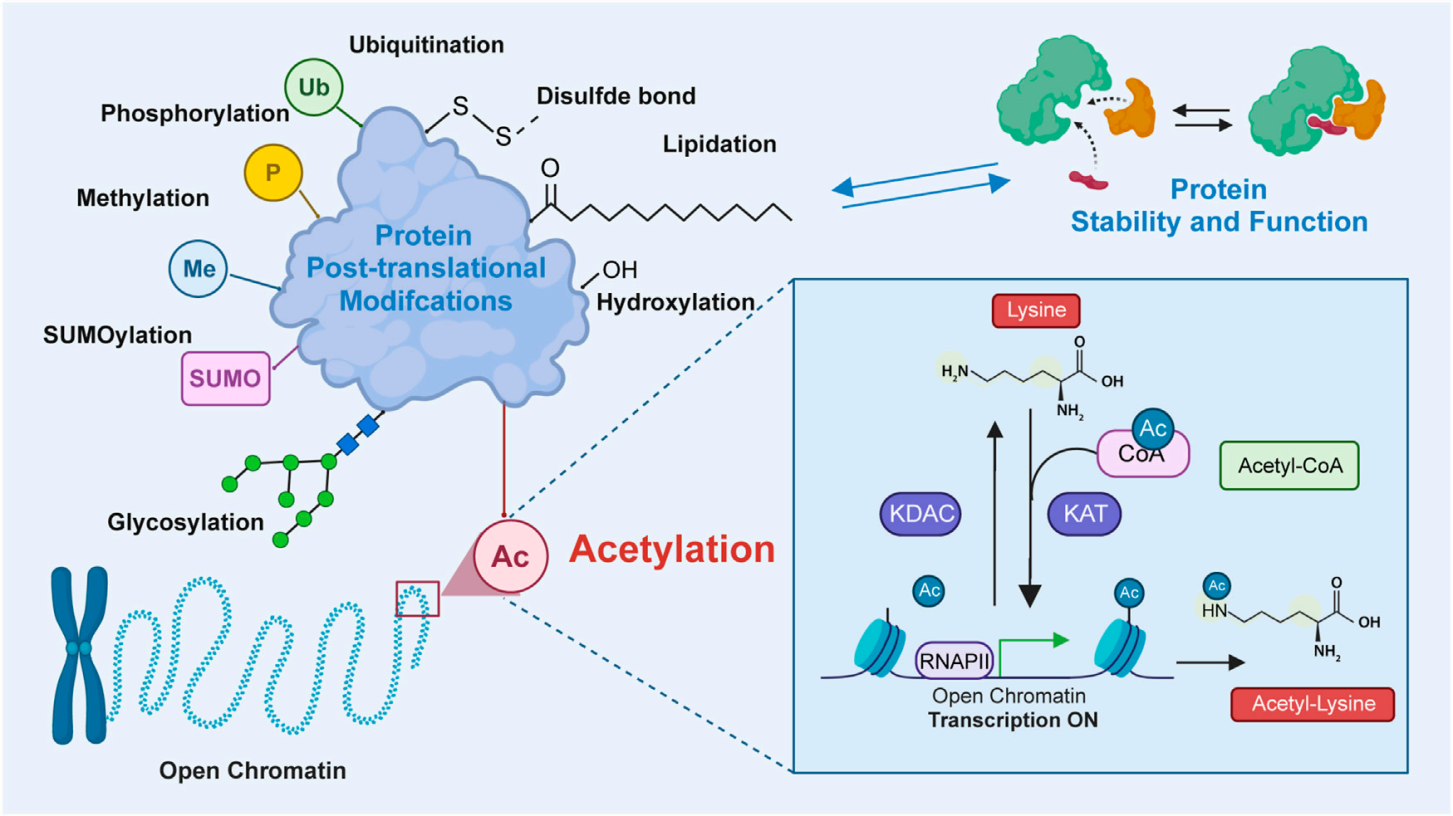
The process of lysine acetylation modification on proteins. In this process, acetyltransferase catalyzes the transfer of acetyl groups to specific lysine residues. The source of this acetyl group is mainly from acetyl-CoA. Acetyltransferase transfers acetyl groups to target proteins by recognizing lysine residues on them. Bromodomains recognize and bind to the acetylated substrates to activate transcription. Additionally, deacetylase, which reverses lysine acetylation, catalyzes the removal of acetyl groups and reduces the acetylation of lysine residues in proteins. Lysine acetylation plays an important role in the regulation of diverse protein functions by modulating protein localization, interaction, stability, and transcriptional activity.

Protein acetylation, a post-translational modification (PTM), involves the covalent attachment of an acetyl group to proteins. Generally, the acetyl group is added to the lysine residues or the N-terminus through acetyltransferase-mediated mechanisms or non-enzymatic mechanisms (Christensen et al., 2019; Ree et al., 2018; Bloch and Borek, 1946). Previous studies have demonstrated that protein acetylation mediates diverse physiological and pathological functions by regulating various proteins and signaling pathways (Shen et al., 2015; Kong et al., 2022; Shvedunova and Akhtar, 2022; Liu et al., 2021). Lysine acetylation, which is the most extensively studied PTM, was first identified to be a histone modification by Vincent Allfrey et al., in 1964 (Phillips, 1963). In addition to histones, lysine acetylation has been subsequently identified in several eukaryotic non-histone proteins (Narita et al., 2019; Saxholm et al., 1982). Since the advent of antibody enrichment and mass spectrometry techniques in 2006, various acetylation-related enzymes and their functions have been identified (Verdin and Ott, 2015; Xue et al., 2022; VanDrisse and Escalante-Semerena, 2019; Morales-Tarré et al., 2021). Lysine acetylation is predominantly mediated through the transfer of an acetyl group from acetyl-CoA to the ε-amino side chain of lysine. This process is reversible, which is called lysine deacetylation, a process catalyzed by deacetylases (Marmorstein and Zhou, 2014). The enzymatic mechanisms are primarily mediated by the following two groups of enzymes: lysine acetyltransferases [KATs; previously called histone acetyltransferases (HATs)] and lysine deacetylases [KDACs; also called histone deacetylases (HDACs)] (Vo et al., 2018; Wu et al., 2019). Bromodomain-containing proteins (BRDs) specifically recognize and bind to acetylated lysine via an evolutionarily conserved asparagine residue within the bromodomain (Figure 1) (Del et al., 2019). Recent studies have reported that lysine can also be acetylated through non-enzymatic mechanisms in the mitochondrial matrix.

Lysine acetylation affects various properties and functions of proteins through several regulatory mechanisms. In particular, lysine acetylation affects the stability, subcellular localization, and enzymatic activity of proteins, as well as their interactions with other proteins or DNA (Allfrey et al., 1964; Hebbes et al., 1988; Hebbes et al., 1992; Li et al., 2020). This modification can alter protein function and regulate cellular processes. Thus, lysine acetylation is the most crucial regulatory mechanism in various biological processes, such as DNA repair, chromatin remodeling, cell cycle progression, and cell apoptosis, which are essential for the development and progression of cancer (Kumar et al., 2016; Ding et al., 2022; You et al., 2012). Lysine acetylation is dysregulated in diverse cancer types, contributing to aberrant protein function and disruption of physiological cellular processes (Ramaiah et al., 2021; Kaypee et al., 2016; Jain and Barton, 2017). For example, the upregulated levels of lysine acetylation in some proteins involved in cell cycle regulation can result in uncontrolled cell proliferation, a hallmark of cancer. An understanding of the regulatory mechanisms of lysine acetylation will provide valuable insights into the development and progression of cancers.

Digestive tract cancers encompass a broad spectrum of malignancies that affect various organs within the gastrointestinal tract. These cancers can manifest as diverse types of cancer, such as esophageal cancer (EC), gastric cancer (GC), colorectal cancer (CRC), liver cancer, and pancreatic cancer (PC) (Sung et al., 2021; Ferlay et al., 2018; Smyth et al., 2020). The symptoms of digestive system tumors may vary depending on the affected organ. However, most digestive system tumors are not easily detectable in the early stages (Issa and Noureddine, 2017; Ladabaum and Mannalithara, 2016). Currently, some success has been achieved in the treatment of digestive tract cancers (Kaźmierczak-Siedlecka et al., 2022; Kronig et al., 2023; Rao et al., 2022). In particular, targeted therapy and immunotherapy have emerged as promising therapeutic modalities for advanced and refractory digestive tract cancers (Chen et al., 2021; Zhao et al., 2023; Patel and Cecchini, 2020; Xue et al., 2023; Ai et al., 2023). However, the efficacy of treatments for advanced metastatic tumors is suboptimal (Galun et al., 2017; Wang et al., 2019). Additionally, early diagnosis of some digestive tract cancers, such as PC, is challenging. Thus, most patients are diagnosed with advanced-stage cancer and exhibit poor prognosis (Klein, 2021; Stoffel et al., 2023; Cai et al., 2021). As there is a continuous demand for comprehensive treatment modalities and personalized treatment strategies, the pathogenesis of digestive tract cancers must be elucidated.

Previous studies have elucidated the correlation of the dysregulated KATs, KDACs, and BRDs (involved in lysine acetylation) with the progression of various digestive tract cancers. Thus, lysine acetylation is a potential therapeutic target for digestive tract cancers (Table 1) (Li and Seto, 2016; Zhao et al., 2022; Jin et al., 2021; Carbajo-Pescador et al., 2013; Cougot et al., 2007; Yang et al., 2023). This review provides an overview of the components and reversible modification process of lysine acetylation. Next, the regulatory mechanisms of lysine acetylation that contribute to the development of digestive tract cancers have been discussed. Additionally, advances in drug development and identification of prognosis biomarkers targeting lysine acetylation in digestive tract cancers have been summarized. Finally, potential future research directions and challenges associated with targeting lysine acetylation in digestive tract cancers have been discussed.

**TABLE 1:** Roles and mechanisms of lysine acetylation in the development of digestive tract cancers.

Cancer	Type of acetylases	Regulator	Role	Related target	Underlying mechanisms	Functions	References
Hepatocellular carcinoma	Lysine acetyltransferase	KAT6A	Cancer-promoting	H3K9	—	Inhibit senescence	Baell et al. (2018)
Hepatocellular carcinoma	Lysine acetyltransferase	KAT6A	Cancer-promoting	H3K23	Matrix stiffness/TRIM24/SOX2	Promote cell proliferation, colony formation, and tumor growth	Zhao et al. (2022)
Hepatocellular carcinoma	Lysine acetyltransferase	KAT6A	Cancer-promoting	H3K23	YAP	Promote sorafenib resistance, and cell proliferation	Jin et al. (2021)
Hepatocellular carcinoma	Lysine acetyltransferase	KAT6B	Cancer-restraining	—	—	—	Jiang et al. (2021)
Hepatocellular carcinoma	Lysine acetyltransferase	CBP/p300	Cancer-promoting	VEGF	STAT3/HIF1α	Promote angiogenesis	Carbajo-Pescador et al. (2013)
Hepatocellular carcinoma	Lysine acetyltransferase	CBP/p300	Cancer-promoting	—	—	Inhibit cell proliferation, and invasion	Inagaki et al. (2016)
Hepatocellular carcinoma	Lysine acetyltransferase	CBP/p300	Cancer-promoting	CREB	HBx	Promote cell transformation	Cougot et al. (2007)
Hepatocellular carcinoma	Lysine acetyltransferase	p300	Cancer-promoting	—	—	—	Li et al. (2011)
Hepatocellular carcinoma	Histone deacetylase	HDAC1, HDAC2, and HDAC3	Cancer-promoting	—	—	Promote cell proliferation, migration, invasion, colony formation, and 3D spheroid formation	Han et al. (2020)
Hepatocellular carcinoma	Histone deacetylase	HDAC1, HDAC2, and HDAC3	Cancer-promoting	PD-L1, and Class I MHCA	Beclin1	inhibit cell autophagy	Roberts et al. (2020)
Hepatocellular carcinoma	Histone deacetylase	HDAC6	Cancer-restraining	NF-κB, Beclin 1, and p62/SQSTM	miR-221	Promote cell autophagy	Bae et al. (2015)
colorectal cancer	Lysine acetyltransferase	KAT6A	Cancer-promoting	H3K23	DANCR/TRIM24/YAP	Promote cell proliferation, and colony formation	Xie et al. (2020)
colorectal cancer	Lysine acetyltransferase	KAT6A	Cancer-promoting	H3K23	DANCR	Promote cell cycle progression, and cell proliferation	Lian et al. (2020)
colorectal cancer	Lysine acetyltransferase	KAT6B	cancer-promoting	STAT3	Circ-MALAT1/miR-506–3p/	Promote cell proliferation, migration, and EMT	Yang et al. (2023)
colorectal cancer	Lysine acetyltransferase	CBP/p300	cancer-promoting	KLF5	CCL7/CXCL5	Promote cell proliferation, and metastasis	Xu et al. (2022)
colorectal cancer	Lysine acetyltransferase	HDAC1, and CBP/p300	Cancer-promoting	—	—	—	Ishihama et al. (2007)
gastric cancer	Lysine acetyltransferase	KAT6B	Cancer-restraining	—	miR-4513	inhibit cell proliferation, invasion, and EMT	Ding et al. (2019)
gastric cancer	Histone deacetylase	HDAC2	Cancer-promoting	CITED2		Promote anthracycline chemoresistance, and cell proliferation	Regel et al. (2012)
gastric cancer	Histone deacetylase	HDAC	—	—	—	Promote immunotherapy efficacy	Lin et al. (2023)
gastric cancer	Histone deacetylase	HDAC1, HDAC2, and HDAC3	Cancer-promoting	B7-H1	IFN-γ	Promote immune evasion	Deng et al. (2018)
esophageal cancer	Lysine acetyltransferases	CBP/p300	Cancer-promoting	linc00460	—	Promote cell proliferation, and cell cycle	Liang et al. (2017)
esophageal cancer	Histone deacetylase	HDAC	Cancer-promoting	—	FB 1, and PI3K/Akt	Promote cell proliferation, and migration	Yu et al. (2021)
esophageal cancer	Histone deacetylase	HDAC	Cancer-promoting	—	ROS, and IRE1α/JNK	Promote cell proliferation, and migration	Jian et al. (2022)
pancreatic cancer	Lysine acetyltransferase	CBP/p300	Cancer-promoting	IDH1, FASN, and MTHFD1	—	Promote cell metabolism, and cancer progression	Zheng et al. (2023)
pancreatic cancer	Lysine acetyltransferase	CBP/p300	Cancer-promoting	VEGF	STAT3/HIF1α	Promote angiogenesis	Gray et al. (2005)
pancreatic cancer	Histone deacetylase	HDAC5	—	p65	PD-L1	inhibit cell susceptibility to immunotherapy	Zhou et al. (2022)
pancreatic cancer	Histone deacetylase	HDAC1	Cancer-promoting	SRF, FOXM1, and LIF	—	Promote fibrous tissue proliferation, and tumor formation	Liang et al. (2023)
pancreatic cancer	Histone deacetylase, and BET	HDAC1, and BRD4	Cancer-promoting	—	—	Promote cell proliferation	He et al. (2020)

HDAC, histone deacetylase; BRD, bromodomain-containing protein; HBX, hepatitis B virus X protein; EMT, epithelial-mesenchymal transition; NF, nuclear factor; PD-L1, programmed death ligand 1; VEGF, vascular endothelial growth factor; CREB, cyclic AMP-response element-binding protein.

## Components and dynamic processes of lysine acetylation

Acetylation of lysine residues is a reversible PTM process with critical roles in modulating protein functions and cellular processes. The dynamic balance between lysine acetylation and deacetylation involves multiple components, including enzymes (KATs and KDACs) and KAT substrate recognition proteins (BRDs). The functions of lysine acetylation in regulating various cellular processes are diverse and context-dependent. The dynamic nature of lysine acetylation is a key feature that allows organisms to respond to both intracellular and extracellular signals, leading to rapid and reversible modifications of proteins. This dynamic process enables cells to fine-tune protein functions and gene expression to adapt to constantly changing environmental conditions. In this section, a detailed overview of KATs and KDACs involved in lysine acetylation, as well as the BRDs that specifically recognize acetylated lysine residues, has been provided.

## KATs

Based on the amino acid sequence homology, KATs are currently classified into the following three major families: the MYST (MOZ, Ybf2/Sas3, Sas2, and TIP60) family, the p300/cyclic AMP-response element (CRE)-binding protein (CREB)-binding protein (CBP) family, and the GCN5/PCAF (general control nonderepressible 5 and its human ortholog PCAF; also known as KAT2A/KAT2B) family (Roth et al., 2001; Yang et al., 2020; Simon et al., 2016; Yang, 2004; Wapenaar and Dekker, 2016). Among these families, the p300/CBP family members without the conserved motif found in other acetyltransferases comprise p300 and CBP (also known as KAT3B/KAT3A) and are mainly localized to the nucleus and cytoplasm. Additionally, the p300/CBP family members are involved in the targeted acetylation of specific chromatin domains (McIntyre et al., 2019; Ogryzko et al., 1996; Dutta et al., 2016).

## KDACs

In mammals, the KDAC superfamily comprises 11 zinc-dependent HDACs and 7 nicotinamide adenine dinucleotide (NAD^+^)-dependent sirtuins (Shvedunova and Akhtar, 2022; Menzies et al., 2016; Ellmeier and Seiser, 2018). These two subgroups of KDACs regulate transcription. The function of HDACs is dependent on zinc ion (Zn^2+^) as a coenzyme, while that of sirtuins is dependent on NAD^+^ as a cofactor for the removal of acetyl groups (Penney and Tsai, 2014). Based on sequence similarity, KDACs can be divided into 4 different classes. Class I KDACs, which comprise four members (HDAC1, HDAC2, HDAC3, and HDAC8), are highly similar to the yeast protein RPD3 (Moreno-Yruela et al., 2022). Class II KDACs comprise Class IIa (HDAC4, HDAC5, HDAC7, and HDAC9) and class IIb (HDAC6 and HDAC10) enzymes. HDAC11 is a unique member of class IV KDACs. Sirtuins belong to class III KDACs with seven members (SIRT1 to SIRT7) and exhibit homology to yeast protein Sir2.

## BRDs

In addition to KATs and KDACs, lysine acetylation is also regulated by KAT substrate recognition proteins (Gong et al., 2016; Zaware and Zhou, 2019). BRDs with approximately 110 amino acid structural domains recognize specific acetylated lysine residues on target proteins and recruit KATs or KDACs to regulate their acetylation status. Furthermore, the human bromodomain and extra-terminal (BET) family, which functions as a well-conserved class of transcriptional regulators, has piqued the interest of the scientific community (Filippakopoulos et al., 2010; Ferri et al., 2016; Taniguchi, 2016). The BET family in humans comprises four members (BRD2, BRD3, BRD4, and BRDT), which comprise two N-terminal bromodomains (BD1 and BD2).

These components function together in the process of lysine acetylation and regulate various cellular biological functions, including gene expression, cell cycle regulation, and cell signaling. The interplay between KATs, KDACs, and BRDs has a critical role in regulating diverse epigenetic processes and can be targeted to treat various diseases, including digestive tract cancers (Alleboina et al., 2021; Ackloo et al., 2017).

## Lysine acetylation in digestive tract cancers

**FIGURE 2:**
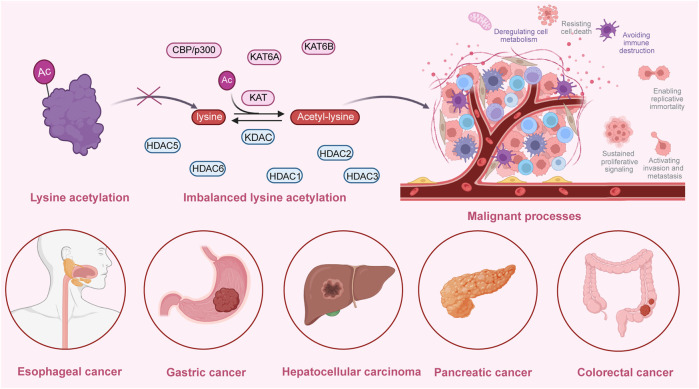
An overview of the dysregulated lysine acetylation in multiple digestive tract cancers. Various lysine acetyltransferases and deacetylases, such as KAT6A, KAT6B, CBP/p300, HDAC1, HDAC2, HDAC3, HDAC5, and HDAC6 are frequently dysregulated in several digestive tract cancers, including hepatocellular carcinoma, colorectal cancer, gastric cancer, esophageal cancer, and pancreatic cancer. Moreover, these dysregulated enzymes exert regulatory effects on the expression and functions of specific target molecules, modulating multiple malignant biological processes during the progression of cancer. These processes include cell proliferation, colony formation, cell metabolism, invasion, migration, autophagy, drug resistance, immune evasion, and angiogenesis.

**FIGURE 3:**
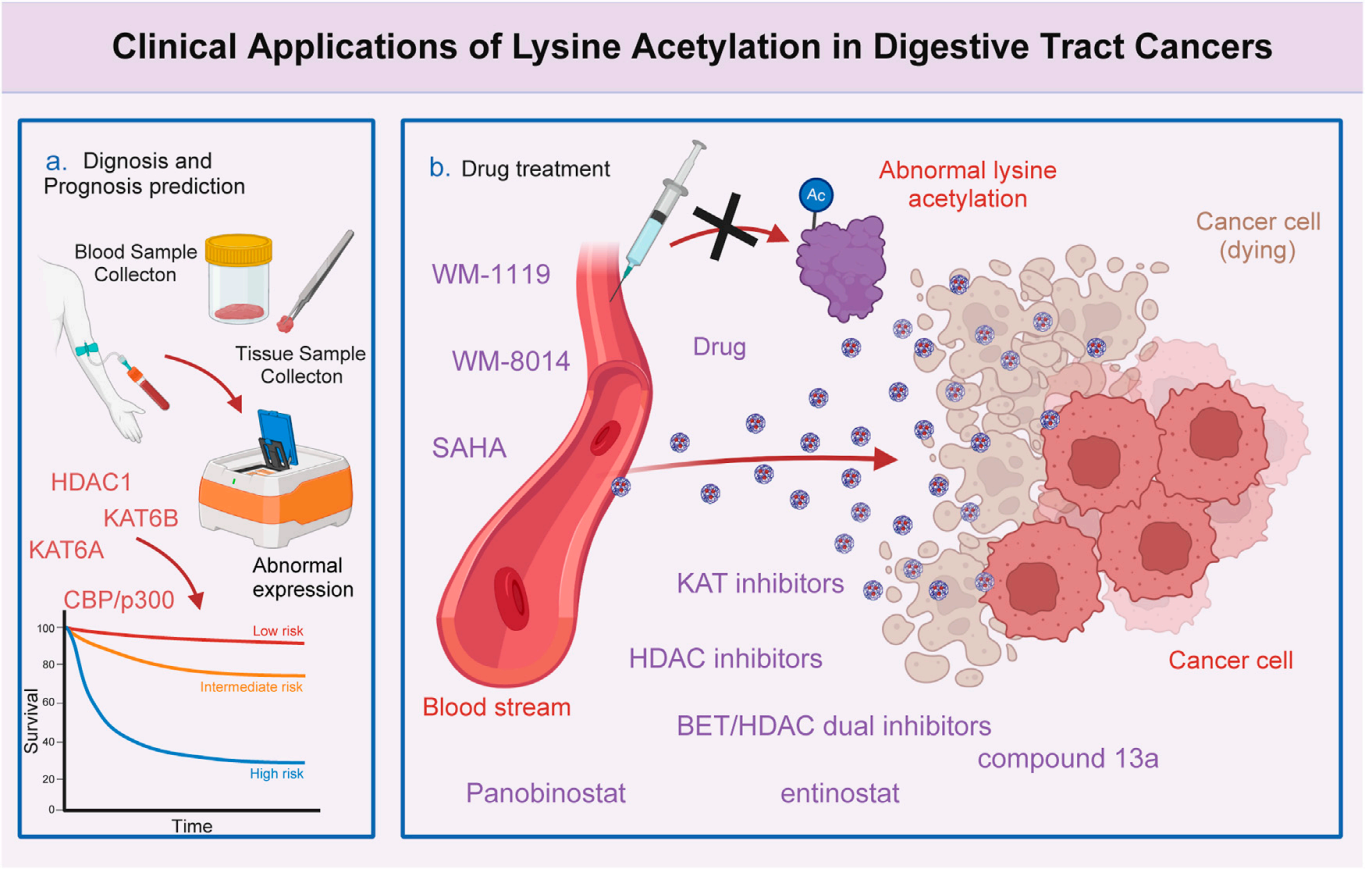
Clinical significance of lysine acetylation in digestive tract cancers. The dysregulation of multiple lysine acetyltransferases and deacetylases, including KAT6A, KAT6B, CBP/p300, and HDAC1, is closely associated with the clinical characteristics and prognosis of patients with digestive tract cancers. This association enables precise and sensitive prediction of patient prognosis by assessing the expression levels of these enzymes in patients with digestive tract cancers. Additionally, several lysine acetylation-targeting agents have been developed. Several promising candidates have shown encouraging efficacy in treating digestive tract cancers. Currently, various KAT and HDAC inhibitors, such as WM-8014, WM-1119, panobinostat, suberoylanilide hydroxamic acid (SAHA), and entinostat, exert effective anti-cancer effects in various models of digestive tract cancer. Furthermore, several bromodomain and extra-terminal domain (BET)/HDAC dual inhibitors are under development, and some drugs, such as compound 13a have exhibited excellent synergistic effects.

Several studies have demonstrated that lysine acetylation is dysregulated in multiple digestive tract cancers, including EC, GC, CRC, liver cancer, and PC. The dysregulation of lysine acetylation and deacetylation actively contributes to the malignant behaviors of digestive tract cancers, such as cell proliferation, migration, and metabolism, stem cell properties, and drug resistance through the regulation of cancer-associated genes and pathways (Figure 2) (Narita et al., 2019; Xu et al., 2014). Additionally, the expression profiles of various lysine acetylation regulators are closely associated with the clinicopathological indices and outcomes of patients with digestive tract cancers, suggesting their potential prognostic values (Figure 3). The regulatory effects of lysine acetylation on cancer-associated genes and pathways provide useful insights into the pathogenesis of digestive tract cancers from the PTM perspective. Thus, emerging inhibitors have been developed and tested in several digestive tract cancers (Table 2). In this section, the aberrant expression and functions of lysine acetylation regulators in diverse digestive tract cancers have been highlighted. Additionally, this section discusses the regulatory mechanisms of these regulators in the development of digestive tract cancers and their clinical applications.

**TABLE 2:** Clinical significance of lysine acetylation in digestive tract cancers.

Type of acetylases	Regulator	Cancer	Expression	Clinical application	Inhibitor	Publication year	References
Lysine acetyltransferase	KAT6A	Hepatocellular carcinoma	—	Therapeutic target	WM-8014	2018	Baell et al. (2018)
Lysine acetyltransferase	KAT6A	Hepatocellular carcinoma	Upregulated	Prognostic predictor	—	2022	Zhao et al. (2022)
Lysine acetyltransferase	KAT6A	Hepatocellular carcinoma	Upregulated	Therapeutic target	WM-1119	2021	Jin et al. (2021)
Lysine acetyltransferase	KAT6B	Hepatocellular carcinoma	Downregulated	Prognostic predictor	—	2021	Jiang et al. (2021)
Lysine acetyltransferase	CBP/p300	Hepatocellular carcinoma	Upregulated	Prognostic predictor	C646	2015	Inagaki et al. (2016)
Lysine acetyltransferase	p300	Hepatocellular carcinoma	Upregulated	Prognostic predictor	—	2011	Li et al. (2011)
Lysine acetyltransferase, and Histone deacetylase	HDAC1, and CBP/p300	Colorectal cancer	Upregulated	Prognostic predictor	—	2007	Ishihama et al. (2007)
Lysine acetyltransferases	KAT6B	Castric cancer	Downregulated	Prognostic predictor	—	2019	Ding et al. (2019)
Histone deacetylase	HDAC1, HDAC2, and HDAC3	Hepatocellular carcinoma	Upregulated	Therapeutic target	Valeric Acid	2020	Han et al. (2020)
Histone deacetylase	HDAC1, HDAC2, and HDAC3	Hepatocellular carcinoma	Upregulated	Therapeutic target	entinostat	2020	Roberts et al. (2020)
Histone deacetylase	HDAC2	Castric cancer	Upregulated	Therapeutic target	Panobinostat	2012	Regel et al. (2012)
Histone deacetylase	HDAC	Castric cancer	—	Predict immunotherapy efficacy	—	2023	Lin et al. (2023)
Histone deacetylase	HDAC1, HDAC2, and HDAC3	Gastric cancer	Upregulated	Therapeutic target	SAHA	2018	Deng et al. (2018)
Histone deacetylase	HDAC	Esophageal cancer	—	Therapeutic target	TSA	2021	Yu et al. (2021)
Histone deacetylase	HDAC	Esophageal cancer	Upregulated	Therapeutic target	CUDC-907	2022	Jian et al. (2022)
Histone deacetylase	HDAC5	Pancreatic cancer	—	Therapeutic target	LMK235	2022	Zhou et al. (2022)
Histone deacetylase	HDAC1	Pancreatic cancer	Upregulated	Therapeutic target	entinostat	2023	Liang et al. (2023)
Histone deacetylase, and BRD	HDAC1, and BRD4	Pancreatic cancer	—	Therapeutic target	compound 13a	2020	He et al. (2020)

SAHA, suberoylanilide hydroxamic acid; TSA, trichostatin A; HDAC, histone deacetylase; BRD, bromodomain-containing protein.

## KATs and digestive tract cancers

According to The Cancer Genome Atlas data analysis, most KATs, including KAT8, KAT5, KAT7, KAT6A, and KAT6B, are frequently mutated in diverse digestive tract cancers, especially in CRC, GC, and EC (Chen et al., 2016; Wang et al., 2011; Ahn et al., 2014; Ng et al., 2022). The levels of lysine acetylation and lysine acetylation regulators are reported to be dysregulated in various digestive tract cancers, suggesting the correlation between lysine acetylation and the pathogenesis of digestive tract cancers (Ding et al., 2019).

Several studies have suggested that KAT6A is upregulated in hepatocellular carcinoma (HCC) tissues and cell lines, suggesting that KAT6A is correlated with the pathogenesis of HCC (Zhao et al., 2022). The upregulation of KAT6A induced by matrix stiffness promotes tumorigenesis during HCC progression, contributing to increased cell proliferation, colony formation, and tumor growth. In Huh7 cells, KAT6A upregulation promotes the acetylation of histone H3 at lysine 23 (H3K23) and its interaction with tripartite motif containing 24 (TRIM24). This modification subsequently activates the expression of SRY-box transcription factor 2 (SOX2), driving HCC tumorigenesis (Zhao et al., 2022). Furthermore, CBP/p300 is upregulated in HCC tissues. The transcriptional complex, which comprises phosphorylated signal transducer and activator of transcription 3 (STAT3), hypoxia-inducible factor 1 alpha (HIF1α), and CBP/p300 under hypoxic conditions, is actively recruited to the promoter region of vascular endothelial growth factor (VEGF)-encoding gene. This complex plays a crucial role in upregulating the transcription of VEGF in HepG2 cells. The upregulation of VEGF promotes tube formation in human umbilical vein endothelial cells (Carbajo-Pescador et al., 2013). The recruitment of hepatitis B virus (HBV) X protein (HBx) to CBP/p300 activates CREB-dependent gene transcription in human HCC cells, promoting HBV-mediated oncogenesis (Cougot et al., 2007). Additionally, KAT6A exerts oncogenic effects in CRC by interacting with long non-coding RNA (lncRNA) DANCR to form functional complexes and upregulating the expression of TRIM24 and cell cycle-related proteins (Lian et al., 2020; Xie et al., 2020). Subsequently, TRIM24 interacts with acetylated H3K23 and further activates the transcription of Yes-associated protein (YAP), promoting cell proliferation, cell cycle progression, and colony formation in LOVO cells (Xie et al., 2020). KAT6B is also upregulated in CRC tissues and cells, facilitating the proliferation, migration, and epithelial-mesenchymal transition (EMT) of CRC cells. Previous studies have revealed that circ-MALAT1 interacts with miR-506–3p, resulting in the upregulation of KAT6B expression. Subsequently, KAT6B enhances the acetylation of *STAT3* in the CRC cell lines (LS513 and SW837 cells). The aberrant upregulation of STAT3 promotes the proliferation, migration, and EMT of CRC cells, highlighting a complex regulatory mechanism underlying the pathological role of lysine acetylation in the progression of CRC (Yang et al., 2023). C-C motif chemokine ligand 7 (CCL7), which is secreted by tumor-associated mesenchymal stromal cells (T-MSCs), stimulates the acetylation of Kruppel-like factor 5 (KLF5) through CBP/P300. This process leads to the upregulation of C-X-C motif chemokine ligand 5 (CXCL5). CXCL5 upregulation in the CRC microenvironment enhances the ability of CRC cells to proliferate and metastasize (Xu et al., 2022). KAT6B, which exerts tumor-suppressive effects in GC, is downregulated in GC cells. *KAT6B* knockdown in HGC27 and MGC803 cells promotes cell proliferation, invasion, and EMT caused by its upstream target miR-4513 (Ding et al., 2019). In PC, STAT3, HIF1α, and CBP/p300 also form a promoter complex to bind to the VEGF-encoding gene and activate its expression under hypoxic conditions. This process promotes PC angiogenesis, growth, and metastasis (Gray et al., 2005). Additionally, in response to the alterations in PC extracellular nutrient availability, CBP/p300, HDAC1, and HDAC3 modulate the dynamic crotonylation levels of the metabolic enzymes isocitrate dehydrogenase 1 (IDH1), fatty acid synthase (FASN), and methylenetetrahydrofolate dehydrogenase 1 (MTHFD1), enhancing PC cell metabolism and tumor progression (Zheng et al., 2023). CBP/p300 is reported to function as a co-activator to drive linc00460 transcription. The upregulated linc00460 promotes cell proliferation and inhibits apoptosis in EC cells (KYSE150 and KYSE450 cells) (Liang et al., 2017).

The expression of lysine acetylation regulators is dysregulated in various types of digestive tract cancers. Several studies have reported a significant correlation between the levels of lysine acetylation regulators and poor prognosis in patients with HCC, GC, EC, PC, and CRC (Ishihama et al., 2007). For example, clinical data suggest that KAT6A upregulation is associated with short survival and aggressive clinical features in HCC, including large tumor sizes, advanced tumor-node-metastasis stages, and advanced Edmondson-Steiner grade (Zhao et al., 2022). Furthermore, p300 upregulation is associated with aggressive characteristics of HCC, such as increased alpha-fetoprotein (AFP) level, large tumor size, multiplicity, poor differentiation, and advanced stage. Thus, p300 expression functions as a strong and independent predictor of HCC survival (Li et al., 2011; Inagaki et al., 2016). Conversely, KAT6B downregulation is associated with worse prognosis, as well as with large tumor size and elevated AFP level, in HCC (Jiang et al., 2021). Kaplan-Meier analysis revealed that KAT6B downregulation independently predicts decreased overall survival in patients with GC (Ding et al., 2019). These findings indicate the potential of lysine acetylation regulators to serve as prognostic factors for digestive tract cancers (Zhang et al., 2022). The elucidation of tumorigenesis mechanisms of lysine acetylation in digestive tract cancers will provide a theoretical foundation for the development of novel therapeutic strategies that directly target lysine acetylation. The identification of specific enzymes involved in lysine acetylation and their associated pathways will provide novel avenues for developing precision medicine strategies targeting lysine acetylation. For example, previous studies have reported that KAT6A is upregulated in sorafenib-resistant HCC clinical samples. Silencing KAT6A in sorafenib-resistant HCC cells decreases the enrichment of H3K23 acetylation and RNA polymerase II at the promoter region of the YAP-encoding gene, effectively suppressing cell proliferation and resistance to sorafenib. Furthermore, the combination of the KAT6A inhibitor WM-1119 and sorafenib markedly decreases the viability of sorafenib-resistant HCC cells, delaying HCC progression (Jin et al., 2021). Additionally, studies on the zebrafish model of HCC revealed that WM-8014 suppresses KAT6A-induced specific acetylation of H3K9 and cellular senescence. Treatment with WM-8014 significantly decreased tumor volume and promoted cell cycle exit and cellular senescence without adversely affecting the growth of healthy hepatocytes (Baell et al., 2018). The inhibition of CBP/p300 using a specific inhibitor (C646) impairs the proliferation, apoptotic sensitivity, and invasion of HCC cells in a dose-dependent manner (Inagaki et al., 2016). These results suggest the therapeutic potential of targeting lysine acetylation and contribute to the rapid development of treatments targeting lysine acetylation (Leaver et al., 2019).

## KDACs and digestive tract cancers

Additionally, KDAC expression is dysregulated in several types of digestive tract cancers, contributing to cancer development and progression. Several KDAC inhibitors have been extensively studied for applications in tumor immunotherapy. Various small molecule HDAC inhibitors have been identified and their potential therapeutic efficacy as a cancer immunotherapeutic has been demonstrated (Cheng et al., 2024). HDAC2 is upregulated in human GC tissues, GC cells, and CEA424/SV40 T-antigen (CEA/Tag) transgenic mice, suggesting that HDAC2 is correlated with GC development (Regel et al., 2012). Previous studies have reported that the HDAC2 inhibitor LBH589 increases the acetylation levels and suppresses the expression of a cluster of anthracycline resistance-conferring genes (e.g. *FOXM1*, *HMGB2*, and *TYMS*) in GC cells (AGS and MKN-45 cells). Further studies on transgenic CEA/Tag mice revealed that the combination of LBH589 and anthracycline drugs upregulates the expression of CBP/p300-interacting transactivator with Glu/Asp-rich carboxy-terminal domain 2 (CITED2), enhancing apoptosis and decreasing the viability of AGS cells (Regel et al., 2012). Additionally, the expression levels of HDACs are correlated with the characteristics of the tumor microenvironment (TME) and the efficacy of immunotherapy in GC. Analysis of HDAC score (HDS), which is based on the expression of HDACs in GC, revealed that patients with a high HDS exhibit increased immune infiltration and improved prognosis (Lin et al., 2023). One study reported that HDACs increase the IFN-γ-induced histone acetylation of the B7 homolog 1 (B7-H1)-encoding gene promoter, enhancing B7-H1 expression and promoting tumor growth in GC. In a mouse model of subcutaneously grafted GC, pretreatment with the HDAC inhibitor suberoylanilide hydroxamic acid (SAHA) suppresses tumor growth by downregulating IFN-γ-induced B7-H1 expression (Deng et al., 2018). Recent studies have demonstrated that valeric acid functions as a novel HDAC inhibitor, exerting broad growth-inhibitory effects against liver cancer by inhibiting cell proliferation, colony formation, cell invasion, and spheroid formation (Han et al., 2020). Additionally, the interaction of the HDAC inhibitor entinostat with lenvatinib results in the inactivation of mammalian target of rapamycin complex 1 (mTORC1) and mTORC2, inducing autophagy-mediated liver cancer cell clearance. The enhanced autophagy leads to the upregulation of Beclin 1 (a stress response gene), which subsequently suppresses the levels of HDAC1, HDAC2, and HDAC3. This leads to the downregulation of programmed death ligand 1 (PD-L1) and human major histocompatibility complex class I A (MHCA) (Roberts et al., 2020). However, the downregulation of HDAC6 in liver cancer promotes autophagy-induced Hep3B cell death, exerting a tumor-suppressive effect. During hepatocarcinogenesis, miR-221 serves as a potent inhibitor of HDAC6, suppressing the levels of the autophagy-related molecules Beclin 1 and p62/SQSTM (p62), a process mediated by activated c-Met signaling in HCC cells. HDAC6 also reversely regulates miR-221 expression by suppressing nuclear factor kappa B (NF-κB) activation (Bae et al., 2015). In PC, HDAC5 upregulation is positively correlated with favorable clinical outcomes. Additionally, HDAC5 induces the phosphorylation of p65 at S311, leading to the inhibition of NF-κB-mediated PD-L1 expression. Silencing HDAC5 in Panc02 cells and treating mouse models with HDAC5 inhibitor LMK235 upregulated PD-L1 expression and increased the susceptibility of cancer cells to anti-PD1 antibody therapy in PC (Zhou et al., 2022). Recent studies have demonstrated that class I HDACs stimulate the transcription of pro-desmoplastic and pro-tumorigenic processes in pancreatic stellate cells (PSCs) and cancer-associated fibroblasts (CAFs) through the regulation of serum response factor (SRF), forkhead box M1 (FOXM1), and leukemia inhibitory factor (LIF) expression. In particular, HDACs play a central role in promoting pro-desmoplastic programs and activating PSCs by upregulating FOXM1 and SRF levels. Additionally, HDACs facilitate LIF expression to enhance the pro-tumorigenic characteristics of CAFs. Studies using genetically engineered mouse models treated with the HDAC inhibitor entinostat have further confirmed that HDAC downregulation inhibits the myofibroblastic transcriptional program, leading to the reversal of stromal activation and tumor progression in PC (Liang et al., 2023). In EC, the environmental contaminant fumonisin B1 (FB1) upregulated HDAC expression and activated the phosphoinositide 3 kinase (PI3K)/protein kinase B (Akt) signaling pathway, promoting cell proliferation and migration during esophageal carcinogenesis. The HDAC inhibitor trichostatin A (TSA) and myriocin (ISP-1) repress FB1-induced HDAC expression and inhibit carcinogenesis in human esophageal epithelial cells (Yu et al., 2021). Additionally, dual PI3K-HDAC inhibitor CUDC-907 has been developed to target the elevated activation of the PI3K-Akt pathway and the expression of HDACs in various types of EC cells. Studies using KYSE-450 xenograft nude mouse models have indicated that CUDC-907 inhibits the proliferation, invasion, and migration of EC cells by promoting reactive oxygen species (ROS)-inositol requiring enzyme 1 α (IRE1α)-Jun N-terminal kinase (JNK)-mediated cytotoxic autophagy (Jian et al., 2022). These studies indicate the therapeutic potential of HDAC inhibitors in digestive tract cancers. HDAC inhibitors potentially exert therapeutic effects on digestive tract cancers by modulating cellular signaling pathways and regulating the TME.

## BRDs and digestive tract cancers

BRD dysregulation is reported to be associated with the tumorigenesis and progression of digestive tract cancers. Multiple BRDs recognize and bind to acetylated lysine residues of proteins, regulating the fundamental transcription mechanism. Studies on various disease models have revealed that targeting BET is a potent epigenetic therapeutic strategy (Schwalm and Knapp, 2022). The inhibition of the BET family members using small molecules or specific inhibitors can disrupt the interaction between BET proteins and acetylated histones, suppressing the expression of oncogenes and promoting the activation of tumor suppressor genes. This targeted epigenetic therapy has shown encouraging results in preclinical studies and early-phase clinical trials for digestive tract cancers, including CRC, GC, and EC. For example, the combination of the pan-BET inhibitor JQ1 and HDAC inhibitors (such as SAHA and CI994) exhibits a strong synergistic therapeutic effect on advanced PC, inducing cell apoptosis, reducing tumor volume, and decreasing relapse rate (He et al., 2020; Mazur et al., 2015; Zhang et al., 2020; Seyfinejad and Jouyban, 2022; Shahrajabian and Sun, 2023). Based on the excellent synergistic effect of BET and HDAC inhibitors on PC, several novel BET/HDAC dual inhibitors are being developed. Among these inhibitors, compound 13a exerts balanced inhibitory effects against both BRD4 and HDAC1 in Capan-1 PC xenograft models (He and Tang, 2020). Further studies are needed to determine the efficacy of this approach. However, these findings suggest that the anti-cancer efficacy of the combination of BET and HDAC inhibitors is higher than that of monotherapy (Akhtar et al., 2023; Li et al., 2024).

## Conclusion

Lysine acetylation has been demonstrated to play a major role in the development and progression of various diseases, especially digestive tract cancers. This dynamic and reversible process regulates protein functions and further contributes to the modulation of complex cellular processes involved in tumorigenesis. However, several challenges must be addressed in the analysis of lysine acetylation in digestive tract cancers. The functions and mechanisms of lysine acetylation have been extensively studied. However, the precise role of lysine acetylation in digestive tract cancers has not been elucidated. For example, the exact preferred lysine sites and protein types of enzymes related to lysine acetylation have not been identified. Additionally, the mechanisms underlying the different activities and influences of the same acetylation enzyme in the same cancer type are unclear. A major challenge in the clinical setting is the development of sensitive and specific assays for the detection and quantification of lysine acetylation in cancer tissues. The identification of aberrant acetylation sites is crucial for cancer diagnosis and prognosis prediction. Improving the sensitivity and specificity of these acetylation-related biomarkers for clinical applications is challenging. Furthermore, the effectiveness and safety of KDAC inhibitors are major hurdles to their widespread application in the treatment of digestive tract cancers. Thus, future studies must focus on the precise mechanisms of lysine acetylation in the development and progression of digestive tract tumors. Additionally, large-scale clinical trials are required for the development of epigenetic drugs targeting lysine acetylation and the validation of their efficacy and safety. Continued investigation into the functions and mechanisms of lysine acetylation will provide valuable insights into the complex regulatory processes of lysine acetylation involved in tumorigenesis and may lead to the development of novel diagnostic and therapeutic strategies for digestive tract cancers.
